# Supplementary material for: Exploring the Frozen Armory: Antiphage Defense Systems in Cold-Adapted Bacteria with a Focus on CRISPR-Cas Systems
Source: Microorganisms. 2024 May 20;12(5):1028. doi: 10.3390/microorganisms12051028 (PMC11124354; doi:10.3390/microorganisms12051028)
Supplement: Supplementary file 1 [file microorganisms-12-01028-s001.zip › Readme_SupplemenaryFiles.pdf]

# Readme describing the Supplementary Files for the manuscript: Exploring the frozen armory: Antiphage defense systems in cold-adapted bacteria with focus on CRISPR-Cas systems

Greta Daae Sandsdalen[1], Animesh Kumar[1], Erik Hjerde[1]*

[1] Department of Chemistry, UiT the Arctic University of Norway, Tromsø, Norway
* Correspondence: erik.hjerde@uit.no

This file describes the contents of the Supplementary Files.

**Supplementary File S1**

Contains Supplementary Figures S1 and S2.

Figure S1: Phylogenetic distribution of dataset.
Figure S2: Distribution of antiphage defense systems across prokaryotic family.

**Supplementary File S2**

Table S1: Cold-adapted bacteria used in this study (938 genome accession ID) with corresponding database and GTDB taxonomy.

List of CRISPR Classification used in this study

List of Bacterial defense system and its subtypes

**Supplementary File S3**

This Excel file contains output files from PADLOC analysis and data extracted from this file used in further analysis and figures. Data is systemized in sheets within the file.

Output_PADLOC: Output file from PADLOC analysis. Inclusion criteria for further analysis: E-value <0.01; HMM coverage >0.8; Target coverage >0.8. System named "DMS_other" refer to predicted genes belonging to a defense module not further classified, encoding a potential transcription factor with an unknown target. All PADLOC output accession IDS are cross-linked with respective GTDB taxonomy.

Output_cctyper: Results from CRISPRCasTyper analysis. subtype probability >0.75

PADLOC_cctyper_combined_count: Results from PADLOC analysis and cctyper analysis combined, (after applying filters for inclusion criteria)

PADLOC generated CRISPR predictions were not included in further analysis. PADLOC detects CRISPR arrays with a customized version of CRISPRDetect. In this study we were interested in systems including both cas gene clusters and CRISPR arrays, within close vicinity. This is detected using CRISPRCasTyper.

The file also includes available metadata regarding isolation environments, host associations and oxygen tolerance of bacterial strains. This file was used for further analysis in R.

System_percentage_of_genomes: List of percentage of genomes in our dataset which contains the different antiphage defense systems. Data used to make Figure 1.

systems_heatmap_phylum: Frequency of each antiphage defense system within bacterial phylum. Data used to make Figure 2.

systems_heatmap_family: Frequency of each antiphage defense system within bacterial family. Data used to make Figure S2.

**Supplementary File S4**

Output obtained after running CRISPRCasTyper on downloaded bacterial genomics sequences. Data is systemized in sheets within the file.

cas_operons_orphan.tab: Orphan Cas operons

cas_operons_putative.tab: Putative Cas operons

CRISPR_Cas: All CRISPR arrays

crisprs_near_cas.tab: All CRISPRs part of CRISPR-Cas loci

crispr.gff: All CRISPR

crisprs_orphan.tab: Orphan CRISPRs

**Supplementary File S5**

This Excel file contains Supplementary Tables S2-S4. Tables are systemized in sheets within the file.

Table S2: Predicted CRISPR-Cas systems with Cas9, Cas12a, Cas13a and Cas13b effector endonucleases in cold-adapted bacteria.

Predicted with CRISPRCasTyper. Inclusion filter: complete system with cas operon and CRISPR array, subtype probability >0.75. Cas9 (51 genomes), Cas12a (2 genomes), Cas13a (2 genomes) and Cas13b (3 genomes) with corresponding accession IDs, genomic position of system and host taxonomy (GTDB).

Table S3: Predicted prokaryotic argonaute (pAgo) proteins in cold-adapted bacteria.

PADLOC classifies pAgo systems into: Type I, Type II, solo. Further analysis should be performed to reclassify into short, long etc. 51 predicted pAgo proteins with corresponding accession IDs (47), system, genomic position and host taxonomy (GTDB). Inclusion filter: HMM and Target coverage >0.8, E-value <0.01 and pAgo protein present.

Table S4: Predicted bacterial retron systems in cold-adapted bacteria.

PADLOC classifies retron systems into: Type I - XIII. Further analysis should be performed to reclassify according to current names and locate corresponding ncRNAs. 83 predicted RT proteins with corresponding accession IDs (81), system, genomic position and host taxonomy (GTDB). Inclusion filter: HMM and Target coverage >0.8, E-value <0.01 and retron RT protein present. Excluded systems with RT-effector fusions.
