# Supplementary material for: Exploring the Frozen Armory: Antiphage Defense Systems in Cold-Adapted Bacteria with a Focus on CRISPR-Cas Systems
Source: Microorganisms. 2024 May 20;12(5):1028. doi: 10.3390/microorganisms12051028 (PMC11124354; doi:10.3390/microorganisms12051028)
Supplement: Supplementary file 1 [file microorganisms-12-01028-s001.zip › Supplementary File S1.pdf]

Supplementary File S1: Supplementary Figures

Supplementary Figure S1: Phylogenetic distribution of dataset
Supplementary Figure S2: Distribution of antiphage defense systems across prokaryotic family

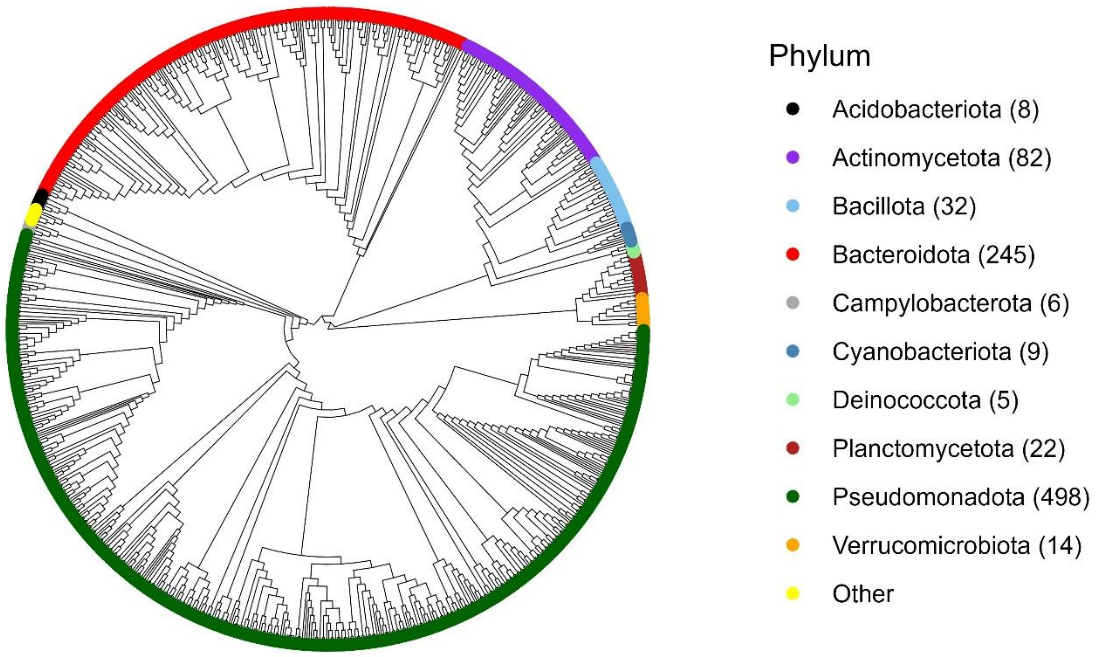

Figure S1. Phylogenetic distribution of cold-adapted bacteria within our dataset, highlighting the top 10 phyla. The number of genomes pertaining to each phylum is indicated in parentheses. The phylogenetic tree is based on GTDB taxonomy classification.

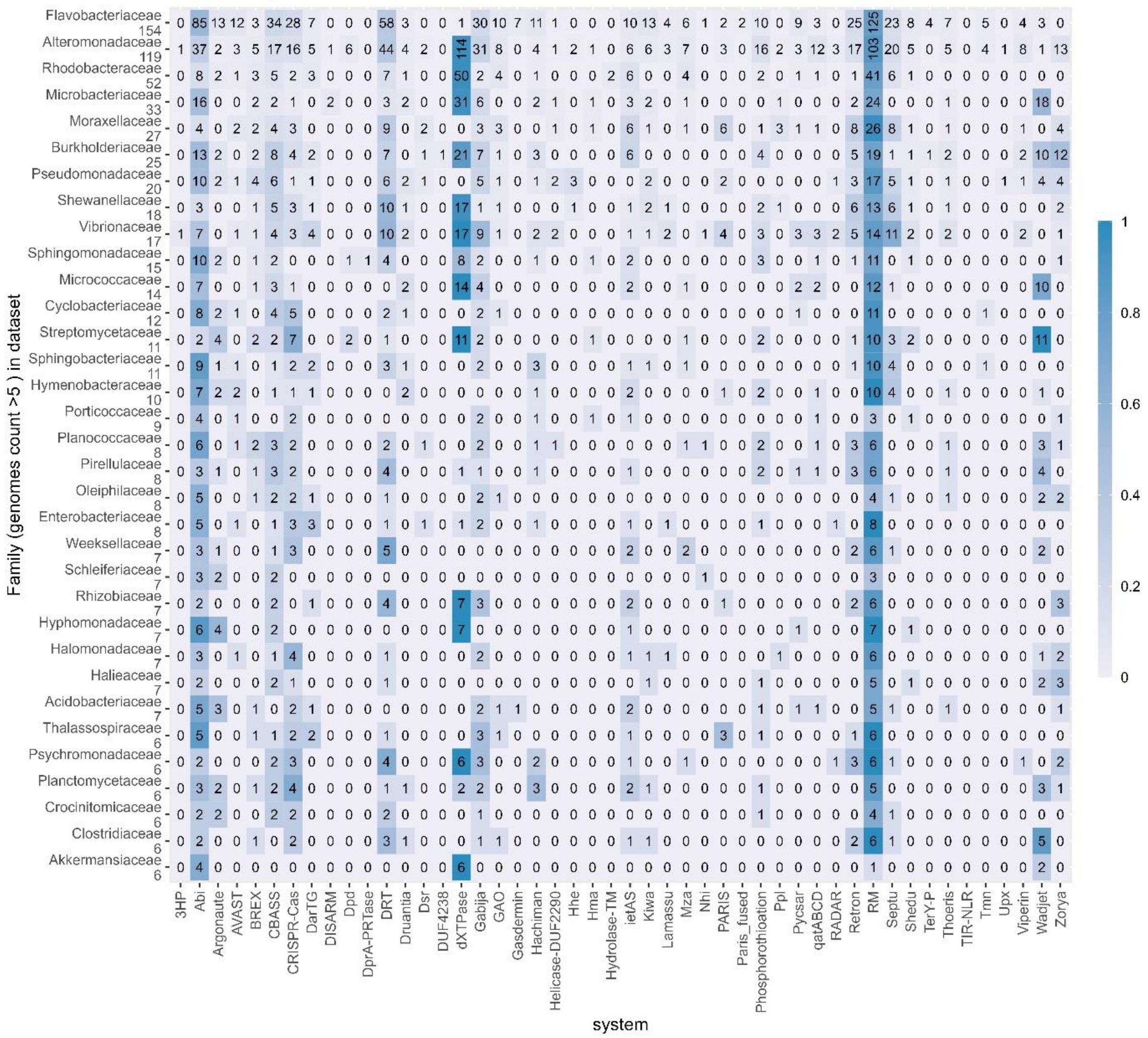

Figure S2. Distribution of systems across prokaryotic family. Only phylogenetic groups with more than 5 genomes are represented. The number of genomes in the dataset is represented under each family name. The heatmap illustrates the system frequency within each phylogenetic group (per row), with a color legend on the right. The absolute number of genomes encoding a particular system is specified in each cell.
